# Colon capsule endoscopy compared with conventional colonoscopy in patients with colonic diverticulitis: a randomized controlled superiority trial on patient-reported outcomes

**DOI:** 10.1055/a-2695-6904

**Published:** 2025-10-17

**Authors:** Camilla Thorndal, Benedicte Schelde-Olesen, Lasse Kaalby, Ulrik Deding, Maja Mi Thygesen, Rene Depont, Lea Østergaard Hansen, Per Vadgaard Andersen, Gunnar Baatrup, Anastasios Koulaouzidis, Thomas Bjørsum-Meyer

**Affiliations:** 1 11286 Department of Surgery, Odense University Hospital, Odense, Denmark; 2 11286 Department of Sugery, Odense University Hospital, Odense, Denmark; 3 6174 Department of Clinical Research, University of Southern Denmark, Odense, Denmark; 4 Department of Gastroenterology, Pomeranian Medical University, Szczecin, Poland; 5 11286 Department of Medicine, Odense University Hospital, Odense, Denmark

## Abstract

**Background:**

In the Danish health care system, follow-up colonoscopy is standard after a colonic diverticulitis episode in order to exclude malignancy. Colon capsule endoscopy (CCE) is a diagnostic alternative to colonoscopy. This study compared patient-reported outcomes of CCE versus colonoscopy after diverticulitis episodes.

**Methods:**

A randomized controlled trial was conducted in patients with computed tomography-verified diverticulitis from Odense University Hospital. Patients were randomized to either CCE or colonoscopy 4–6 weeks after discharge. The primary outcome was patient-reported experienced physical and mental discomfort related to the procedures. Secondary outcomes were expected physical and mental discomfort, examination preference, proportion of complete examinations, and frequency of polyps and colorectal cancer.

**Results:**

159 patients were randomized, with 148 receiving their allocated intervention and 83 completing the questionnaires. Demographic data were comparable between the two groups. No adverse events were observed. Patients expected greater physical and mental discomfort with colonoscopy than with CCE. However, no significant difference was found in experienced physical and mental discomfort between CCE and colonoscopy. For hypothetical future events, 49% of patients would prefer CCE, 13% would prefer colonoscopy, and 38% did not know. Complete examinations were reported for 84% of CCEs and 92% of colonoscopies. No malignant lesions were found.

**Conclusions:**

CCE was a safe follow-up procedure after a diverticulitis episode. CCE was preferred to colonoscopy by most patients who reported their experience. No difference was observed regarding experienced physical and mental discomfort between the groups completing the questionnaires.

## Abstract (de)

**Graphical Abstract:**

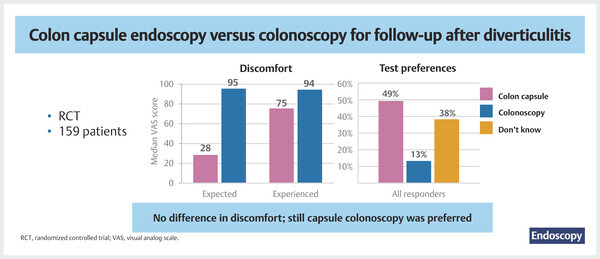

## Introduction

Diverticulosis is a common disease initially thought to affect elderly individuals, with a prevalence of 70% in people aged >70 years and an increasing prevalence with age 1 2 . However, a growing number of younger individuals are affected by the disease 3 , and the lifetime prevalence is estimated to be 72% 4 , making diverticula the most common lesion found incidentally on routine colonoscopy 5 . Nonetheless, it is estimated that only about 20% of individuals will develop symptoms in their lifetime, and <5% will progress from colonic diverticulosis to diverticulitis 6 7 . Yet, this condition can lead to hospitalization and potentially life-threatening complications for patients, such as perforation and bleeding 1 . Furthermore, the economic burden on health care services is significant, although most likely underestimated due to a substantial number of uncomplicated cases being managed outside hospitals 8 9 10 . When patients are admitted to the hospital, diverticulitis is typically classified by the modified Hinchey classification based on severity as determined by a computed tomography (CT) scan 11 12 13 ; if there are no findings indicating the need for further treatment, the patients are discharged.

**Fig. 1:**
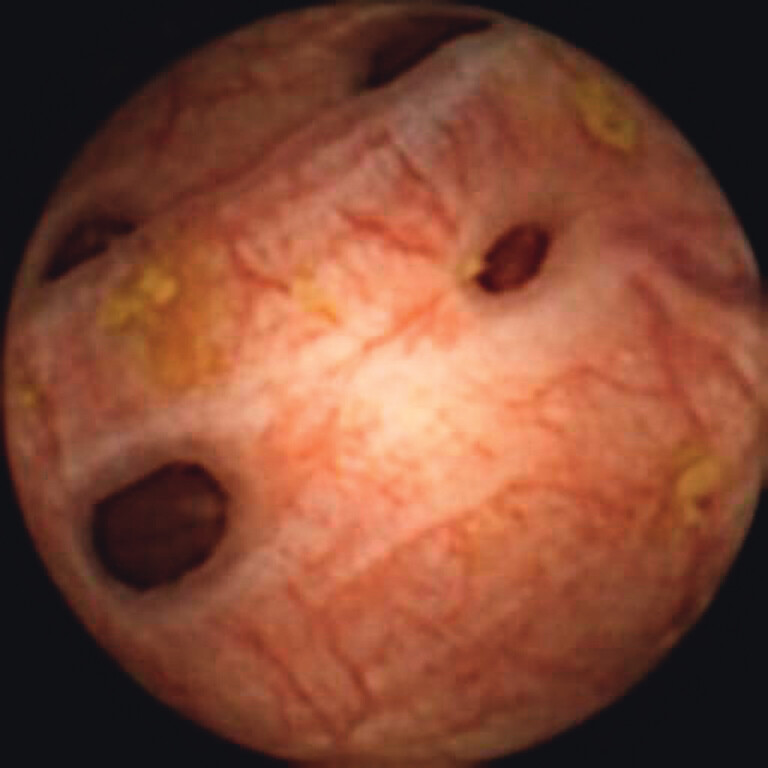
Colon capsule endoscopy image of colonic diverticulitis.

The current standard procedure in the Danish health care system, regardless of the severity of the disease, is that patients are referred for a follow-up colonoscopy 4–6 weeks after discharge following a colonic diverticulitis episode. This aims to confirm the diagnosis of diverticulitis and exclude malignancies. However, this approach is being questioned because studies have found very low rates of colorectal neoplasia in this patient group 14 15 . In colonoscopies performed only for diverticulitis, a low colorectal cancer (CRC) prevalence of 0.17% has been found 16 . An alternative to a follow-up colonoscopy could be colon capsule endoscopy (CCE) ( Fig. 1 ). This procedure has gained increased acceptance in recent years 17 18 , owing to patient preference, a low risk of complications, and the ability to be performed out of hospital 19 20 . However, no research on the use of CCE as a follow-up procedure in patients with diverticulitis has been published.

Therefore, this randomized clinical trial aimed to investigate the patient experience and clinical performance of using CCE compared with colonoscopy as a follow-up examination in patients with diverticulitis.

## Methods

### Trial design

This was a single-center, 1:1 randomized controlled superiority trial conducted at the Surgical and Emergency departments at Odense University Hospital in the Region of Southern Denmark. The trial randomized patients presenting with diverticulitis to either CCE (intervention) or colonoscopy (standard procedure). In cases of incomplete CCE or when CCE identified significant neoplasia, patients of the intervention arm underwent a subsequent colonoscopy.

### Patients

We included patients aged ≥18 years with in-hospital CT-diagnosed diverticulitis (uncomplicated or complicated diverticulitis modified Hinchey grades 1–3). Key exclusion criteria were colonic CT findings requiring biopsy (suspected CRC) or polyp removal, CT-verified stenosis in the gastrointestinal tract, imaging of colonic mucosa within the last 12 months (therefore no indication for renewed endoscopy), cardiac pacemaker, renal insufficiency, pregnancy/breastfeeding, allergies to active substances given in the trial, or inability to provide oral and written informed consent.

### Interventions

The intervention group included patients randomized to CCE. Patients underwent bowel preparation at home, starting 72 hours before CCE. The bowel preparation kit was sent by mail and contained polyethylene glycol (PEG) solutions as well as instructions on how to perform the preparation.

At 72 hours and 48 hours before capsule ingestion, patients took two sachets of PEG and 2 L of water but otherwise remained on a normal diet. The day before and on the day of the procedure, patients ingested 1 L of PEG and 1 L of water with a clear liquid diet.

Before capsule ingestion, patients ingested a 2-mg tablet of prucalopride. After capsule ingestion, patients ingested boosters (sulfate-based saline solution) mixed with water at receiver signals 1–3. The signals were as follows: signal 1 indicated that the capsule had reached the small bowel; signals 2–4 – every 2 hours after signal 1. After the last signal (signal 4), the patients inserted a bisacodyl suppository. The capsule was typically excreted 4–6 hours after ingestion. The CCE investigation was considered complete if the capsule was excreted within the battery lifetime or there was a recording of the anal cushions in patients with acceptable bowel preparation. The bowel preparation was evaluated using the Leighton–Rex grading scale and was determined acceptable if it was fair or better for all colonic segments 21 . If the CCE investigation was incomplete for any reason, the patient was referred for colonoscopy.

If the CCE video showed suspected cancer, more than two polyps, or any number of polyps >9 mm, patients were invited to undergo a diagnostic colonoscopy for biopsies or polyp removal; colonoscopy was performed according to the protocol described below. Any findings on CCE showing no neoplasia or low-risk polyps (defined as 1–2 polyps <10 mm) would elicit no further investigation 22 23 24 ; however, individuals with those results were encouraged to participate in future CRC screening programs if eligible. All CCE videos were assessed by the same expert gastroenterologist, who had experience of evaluating >4000 CCE videos.

The control arm consisted of patients who, following the local guidelines at the Department of Surgery, Odense University Hospital, underwent colonoscopy. The bowel preparation consisted of a PEG+ascorbic acid split-dose regimen, involving two doses: one the day before the procedure and one on the day of the procedure. Five days before the procedure, patients were instructed to eat a diet without seeds and kernels, and the day before the procedure they were instructed not to eat solid foods, and to only drink clear liquids until 2 hours before the procedure, at which point they began fasting. The specific dose of medication and the degree of sedation achieved (conscious sedation using midazolam and pethidine, sedation assisted by anesthesiologists using propofol, or general anesthesia) were registered.

### Outcomes

The primary outcome was patient-reported experienced physical and mental discomfort from colonoscopy and CCE. Secondary outcomes were 1) patient-reported expected physical and mental discomfort associated with colonoscopy and CCE (i.e. examination and the bowel preparation), 2) the discrepancy between the expected and experienced physical and mental discomfort associated with colonoscopy and CCE, 3) the number of complete examinations, 4) the number of patients undergoing CCE who were referred for a subsequent colonoscopy, 5) the prevalence of other abnormal findings, such as polyps and cancers.

To ensure patient safety, complications, adverse events, and investigation quality were monitored continuously by the trial manager during the study.

The expected and experienced physical and mental discomfort scores were measured through questionnaires. Enrolled patients were asked to complete two or three questionnaires before and after their examinations. The first questionnaire (Q1) measured the expected physical and mental discomfort associated with both CCE and colonoscopy. The second questionnaire (Q2) measured the experienced discomfort associated with CCE and was therefore only distributed to those randomized to CCE. The third questionnaire (Q3) measured the experienced discomfort associated with colonoscopy and was therefore only distributed to individuals referred for colonoscopy after their CCE and to those randomized to colonoscopy.

Additionally, patients were contacted by phone by one of the affiliated researchers 7 days after CCE or colonoscopy and asked which examination they would prefer if they needed to undergo a future endoscopic examination, and to state the reason(s) for their choice. All questionnaires were qualitatively validated for the CareForColon2015 trial 25 and then adapted for the current trial.

### Sample size and power considerations

In a previous randomized trial on CCE in a bowel cancer screening population (not yet published) using the same visual analog scale (VAS) as the current trial, the interim analysis of patient-reported outcomes found a mean discomfort score of 18.6 for CCE and 32.2 for colonoscopy (SD 26.5). Applying this difference to the current study (with an SD of 26.5), and with a level of statistical significance of 5% and a power of 80%, a minimum of 60 patients were needed in each group 26 . As this power calculation was performed based on a large sample from the previous trial, we have learnt that the distribution of the experienced discomfort scores tends not to follow a normal or log-normal distribution. With no suitable transformations available, we performed a simulation with 10 000 repetitions of drawing a random sample of 60 from each group from these data. We compared the medians of the experienced discomfort between CCE and colonoscopy for each sample. This enabled us to estimate the power of the median test to detect a difference in medians of at least 17 to be approximately 77% at the two-sided 5% significance level. The simulation was performed using Stata IC 15.1 (StataCorp., College Station, Texas, USA) 27 .

### Statistical analysis

The intention-to-treat 28 population was defined as patients undergoing their allocated randomized procedure. All analyses were for complete cases (i.e. no imputation of missing values was planned). The primary outcome was measured using VAS. The VAS scores were treated as discrete variables with a range of 101 values (0–100, where a score of 0 means no discomfort, and a score of 100 means a very high degree of discomfort). As no normal or log-normal distribution of the recorded VAS scores was present, non-parametric tests were applied. The analyses were modified intention-to-treat analyses, excluding patients who, by their own or physician’s decision, withdrew from the study. For the primary outcome, patients had to have reported their experienced physical and mental discomfort of CCE and colonoscopy, respectively, to be included in the analysis. Univariate comparison for the primary outcome was performed using the Wilcoxon rank sum test in SAS 9.4, TS1M5 (SAS Institute Inc., Cary, North Carolina, USA) 29 . Additionally, continuous ordinal regression models were conducted to test differences in VAS scores 30 31 using the ordinalCont R package in RStudio, R version 4.1.2 (R Foundation for Statistical Computing, Vienna, Austria) 32 33 . The secondary outcomes of expected discomfort, and discrepancy between expected and experienced discomfort, were performed using the Wilcoxon rank sum test, while examination completion rate, and the prevalence of diverticula, polyps, cancers, and other abnormal findings were estimated as proportions and compared between the study arms using the chi-squared test. Multiple statistical testing was not considered.

### Ethics

The Regional Committees on Health Research Ethics for Southern Denmark (ref. S-20210127) and the Danish Data Protection Agency (ref. 22/43235) approved the trial protocol. Our trial protocol paper was published in 2023 34 . All patients provided informed consent.

## Results

### Patients

**Fig. 2:**
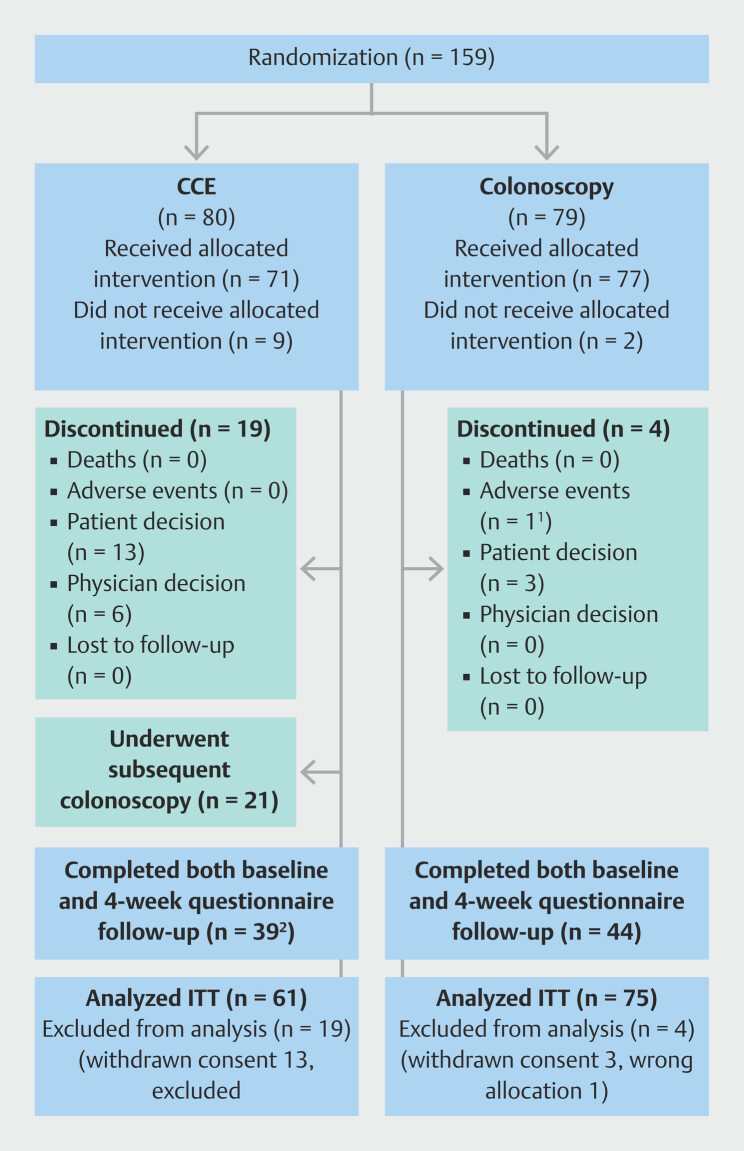
Flow of patients through the study. CCE, colon capsule endoscopy; ITT, intention-to-treat. ^1^ Missed referral, received CCE instead of colonoscopy due to operator error. ^2^ Two individuals did not receive questionnaire 2 due to operator error.

We enrolled patients in the trial between 4 December 2022 and 29 January 2024 at the Surgical Department and Emergency Department of Odense University Hospital in the Region of Southern Denmark. Of the 159 patients who were randomized, 148 received their allocated intervention, and 83 completed the clinical evaluation at week 4, answering either questionnaire 2 and/or 3 ( Fig. 2 ). The demographic and clinical characteristics of the two groups were comparable at baseline ( Table 1 ), except for some imbalance in sex and American Society of Anesthesiologists score.

**Table:** **Table 1** Baseline characteristics of patients randomized to undergo colon capsule endoscopy or colonoscopy after an episode of colonic diverticulitis (intention-to-treat population N = 136).

Characteristics	CCE (n = 61)	Colonoscopy (n = 75)	Total (n = 136)
Female sex, n (%)	39 (64)	54 (72)	93 (68)
Age, mean (SD), years	60.0 (11.1)	60.6 (10.4)	60.3 (10.7)
BMI, kg/m ^2^			
<18.5	0 (0)	1 (2)	1 (1)
18.5–25	16 (31)	15 (25)	31 (28)
>25	36 (69)	43 (73)	79 (71)
*Missing*	9	16	25
ASA score, n (%)			
I	16 (26)	9 (12)	25 (18)
II	45 (74)	66 (88)	111 (82)
CT classification, n (%)			
Uncomplicated diverticulitis ^1^	44 (72)	52 (69)	96 (71)
Pericolic air bubbles or little pericolic fluid without abscess	13 (21)	19 (25)	32 (24)
Abscess			
≤4 cm	4 (7)	4 (5)	8 (6)
>4 cm	0 (0)	0 (0)	0 (0)
Distant air ^2^	0 (0)	0 (0)	0 (0)
Diffuse fluid			
Without distant free air – no colonic perforation	0 (0)	0 (0)	0 (0)
With distant free air – persistent colonic perforation	0 (0)	0 (0)	0 (0)
ASA, American Society of Anesthesiologists; BMI, body mass index; CCE, colon capsule endoscopy; CT, computed tomography. ^1^ Diverticula, thickening of the wall, increased density of the pericolic fat. ^2^ >5 cm from inflamed bowel segment.			

### Primary outcomes

The median VAS score for experienced physical and mental discomfort during the diagnostic investigation was not higher for the patients who underwent colonoscopy (94, interquartile range [IQR] 67) than for those who underwent CCE (75, IQR 71), as the difference was not statistically significant ( *P* = 0.26).

### Secondary outcomes

We observed a significantly higher median VAS score for expected physical and mental discomfort of diagnostic investigations in the patients randomized to colonoscopy than in those assigned to CCE (95 [IQR 71] vs. 28 [IQR 56]; *P* < 0.001) ( Table 2 ). The discrepancy between the median VAS for expected and experienced physical and mental discomfort was significantly higher for patients allocated to CCE (39, IQR 72) than those assigned to colonoscopy (4, IQR 46; *P* = 0.002).

**Table:** **Table 2** Outcomes of colon capsule endoscopy and colonoscopy after an episode of colonic diverticulitis in patients completing questionnaires (n = 83), and in all patients (n = 136).

Outcome	CCE	Colonoscopy	*P* value ^1^
**Patients completing the questionnaire(s), n**	**39**	**44**	
Outcomes, VAS, median (IQR)			
Experienced physical and mental discomfort of diagnostic investigation	75 (71)	94 (67)	0.26
Expected physical and mental discomfort of diagnostic investigation	28 (56)	95 (71)	<0.001
Discrepancy between expected and experienced physical and mental discomfort	39 (72)	4 (46)	0.002
**All patients, n**	**61**	**75**	
Outcomes, n (%)			
Complete examinations	51 (84) ^2^	69 (92)	0.13
Patients with polyp findings	20 (33)	21 (28)	0.55
Patients with polyp findings on CCE and on follow-up colonoscopy	7 (11) ^3^	NA	NA
Patients with cancer suspect findings	0 (0)	0 (0)	NA
Patients with cancer findings confirmed by pathology report	0 (0)	0 (0)	NA
Patients with other abnormal findings	9 (15)	23 (31)	0.030
Patients with other abnormal findings on CCE and follow-up colonoscopy	17 (28)	NA	NA
CCE, colon capsule endoscopy; IQR, interquartile range; VAS, visual analog scale. ^1^ Chi-squared test. ^2^ 100% had complete capsule colon transit, but 7 had poor bowel preparation, and 3 had technical errors. ^3^ Out of the 20 with CCE-detected polyps, 14 underwent colonoscopy, of whom 7 had colonoscopy-identified polyps.			

**Fig. 3:**
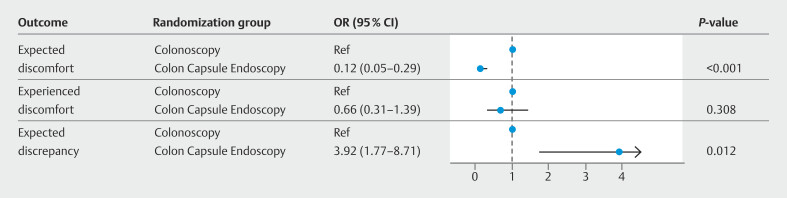
Odds ratios for higher expected physical and mental discomfort, experienced physical and mental discomfort, and discomfort discrepancy compared with the reference (n = 83).

The multivariate models showed a significantly lower probability of higher expected physical and mental discomfort with CCE compared with colonoscopy (odds ratio [OR] 0.12, 95%CI 0.05–0.29), and a considerably higher likelihood of a difference between expected and experienced physical and mental discomfort in the CCE group compared with the colonoscopy group (OR 3.92, 95%CI 1.77–8.71). However, colonoscopy was not associated with a significantly higher probability of experienced physical and mental discomfort than CCE ( Fig. 3 ).

The rate of complete colonoscopy examination was 92% ( Table 2 ). All CCEs (100%) showed complete transit; however, seven were associated with inadequate bowel preparation and three experienced technical errors, giving a complete examination rate of 84%. In total, 33% of patients undergoing CCE and 28% of patients undergoing colonoscopy had polyp findings. Only 11% of patients with polyp findings on CCE had the same polyps detected again on subsequent colonoscopy. Other abnormal findings were present in 31% of patients undergoing colonoscopy and 15% of patients undergoing CCE, while 28% of patients in the CCE group had other abnormal findings on CCE and/or follow-up colonoscopy. There were no findings suspicious for cancer with either procedure.

When asked about preferences for future endoscopic examinations, 49% of patients (48/98 responders) preferred CCE compared with only 13% (13/98) who preferred colonoscopy ( Table 3 ). Furthermore, CCE was preferred to colonoscopy in patients who underwent CCE only (68%) and in patients who underwent colonoscopy only (43%). However, 43% of patients who underwent both investigations would prefer colonoscopy in the future.

**Table:** **Table 3** Preferences for hypothetical future endoscopic examination.

	Preference for future endoscopic examination		
Sample	CCE, n (%)	Colonoscopy, n (%)	Do not know, n (%)
All responders (n = 98)	48 (49)	13 (13)	37 (38)
Responders undergoing only CCE (n = 28)	19 (68)	3 (11)	6 (21)
Responders undergoing only colonoscopy(n = 56)	24 (43)	4 (7)	28 (50)
Responders undergoing both investigations (n = 14)	5 (36)	6 (43)	3 (21)
CCE, colon capsule endoscopy.			

Patients were further asked about the reasons for their future choices. Of the patients who would choose CCE for future endoscopic examinations, the most common explanation for their choice was to experience less physical and mental discomfort, as many described having severe pain, both during and after the colonoscopy. Another common answer was that colonoscopy could be intrusive, often leading to feelings of vulnerability and embarrassment, and issues related to personal privacy as the examination involves an exposure that many patients found to be challenging. Of the patients choosing colonoscopy, most of the explanations for the choice were unfamiliarity with CCE, whereas colonoscopy was more familiar and considered to be the safe and easy choice. A few patients also mentioned the possibility of taking samples during colonoscopy, which is not possible with the less invasive CCE, and seemed especially appealing for those patients with particular concerns. Regardless of the examination, a common response was that the bowel preparation was the worst part of the whole process.

## Discussion

To the best of our knowledge, this is the first study to investigate the performance of CCE compared with colonoscopy as a follow-up procedure after an episode of acute colonic diverticulitis. No statistically significant differences in experienced physical and mental discomfort were found between the two examinations. Expected physical and mental discomfort was lower for patients undergoing CCE compared with those undergoing colonoscopy. Patients preferred CCE except for those undergoing both procedures (n = 14).

One major strength of this study is its design as a randomized controlled trial. However, it is limited by the single-center design and the fact that one reader assessed all CCE videos, which establishes a risk of detection bias. Furthermore, only 55% (39/71) of patients who underwent allocated CCE and 57% (44/77) of patients who underwent allocated colonoscopy completed both baseline and 4-week questionnaires, which presents a risk of selection bias.

In a systematic review from 2021 investigating patient-reported outcomes and preferences for CCE and colonoscopy, the tolerability for CCE was consistently reported as being higher compared with colonoscopy in the included studies 20 . However, no concurrent increase in preference for CCE was shown. One of the main shortcomings of CCE compared with colonoscopy is the lack of interventional options, as patients with neoplastic findings require a subsequent colonoscopy or sigmoidoscopy for polyp removal or biopsy. This knowledge will likely tilt the preference toward colonoscopy. Another drawback of CCE is the need for more extensive bowel preparation, as peri-procedural cleansing of the bowel mucosa with flushing and suction is only possible during colonoscopy. The bowel preparation in the present study commenced 72 hours before the CCE procedure, whereas patients allocated to colonoscopy required only 1 day of preparation before colonoscopy. Despite extensive bowel preparation, CCE failed to meet the completion rate standard for colonoscopy of >90% 35 . However, no statistically significant difference was detected in completion rates for CCE and colonoscopy in the current study (84% vs. 92%, *P* = 0.13).

For decades, it has been debated whether colonoscopy is mandatory after an episode of colonic diverticulitis to rule out an underlying malignancy. In a recent systematic review of endoscopic findings after CT-confirmed colonic diverticulitis, CRC was detected in 2.0% of cases, whereas advanced adenomas were detected in 3.8% 36 . Interestingly, 15% of detected cancers were incidental findings located at sites other than the site of diverticulitis on CT. Furthermore, the OR for CRC in patients with complicated diverticulitis compared with uncomplicated diverticulitis was 9.2 36 .

The prevalence of CRC in patients with colonic diverticulitis is 3–6 times higher than the global population prevalence of 0.3%–0.4%. Besides early diagnosis of CRC, a follow-up colonoscopy offers detection of advanced adenomas and contributes to addressing other disease states such as inflammatory bowel disease, lymphoma, and ischemic colitis. The risk of complications during colonoscopy, although low (<0.5%), needs to be considered to ensure the benefits outweigh the risks. Moreover, in many countries, endoscopy units are overburdened with long waiting lists for colonoscopy.

In that context, CCE is a pertinent modality to rule out malignancy after an episode of colonic diverticulitis. It has a CRC detection rate equivalent to colonoscopy 37 38 , no risk of bleeding or perforation, and can be performed out of hospital in the patient’s home. Larger studies powered to investigate CRC detection rates for CCE in patients after an episode of diverticulitis are needed. Of course, patients with significant lesions and incomplete CCE will need a subsequent colonoscopy. In our study, 34.4% (21/61) of patients were in this category, which needs to be improved by identifying patients at high risk of poor bowel preparation. Moreover, the need for a subsequent colonoscopy can be decreased by a “diagnose-and-leave” approach, meaning that diminutive and small polyps <10 mm detected by CCE are ignored, and the patient is encouraged to participate in existing bowel cancer screening programs.

In conclusion, no difference in experienced physical and mental discomfort was observed between patients randomized to CCE and those undergoing colonoscopy in patients completing questionnaires. CCE was a safe alternative to colonoscopy after an episode of colonic diverticulitis to rule out an underlying malignancy. It was preferred by patients, except for those who needed a subsequent colonoscopy after CCE due to incomplete examination or pathological findings. Bowel preparation for CCE needs to be improved to meet the standards of colonoscopy.
